# Peripheral Anti-Angiogenic Imbalance during Pregnancy Impairs Myogenic Tone and Increases Cerebral Edema in a Rodent Model of HELLP Syndrome

**DOI:** 10.3390/brainsci8120216

**Published:** 2018-12-06

**Authors:** Cynthia Bean, Shauna-Kay Spencer, Mallikarjuna R. Pabbidi, Jamie Szczepanski, Sarah Araji, Sellena Dixon, Kedra Wallace

**Affiliations:** 1 Department of Obstetrics & Gynecology, University of Mississippi Medical Center, Jackson, MS 39216, USA; cinbeanmd@gmail.com (C.B.); sspencer2@umc.edu (S.-K.S.); jszczepanski@umc.edu (J.S.); saraji@umc.edu (S.A.); 2 Department of Pharmacology & Toxicology, University of Mississippi Medical Center, Jackson, MS 39216, USA; mpabbidi@umc.edu; 3 BasePair Program, Murrah High School, Jackson, MS 39216, USA; sldixon@umc.edu; 4 Department of Neuroanatomy and Anatomical Sciences, University of Mississippi Medical Center, Jackson, MS 39216, USA

**Keywords:** edema, pregnancy, myogenic tone, sFlt-1, sEndoglin, tight junction

## Abstract

Using an animal model of hemolysis elevated liver enzymes low platelets (HELLP) that has systemic inflammation and neuroinflammation we wanted to determine if blood brain barrier (BBB) permeability, cerebral edema, vascular tone, and occludin expression were altered in pregnant rats. Anti-angiogenic proteins sFlt-1 and sEng (4.7 and 7 µg/kg/day, respectively) were chronically infused into normal pregnant (NP) rats beginning on gestational day 12 via a mini-osmotic pump. On gestational day 19, blood pressure was measured via a carotid catheter and brains were collected. BBB permeability was assessed in select brain regions from rats infused with 0.5 mg/mL Texas Red Dextran and phenylephrine. Occludin, sFlt-1, and sEng were analyzed via western blot or ELISA. Infusion of sFlt-1 and sEng into NP rats increased hemolysis and liver enzymes, and decreased platelets and led to hypertension. HELLP rats had significant impairment in the myogenic response and increased BBB permeability in the posterior cortex and brainstem. Brain water content in the posterior cortex was increased and sEng protein expression in the brainstem was significantly increased in HELLP rats. The results from this study suggest that a peripheral anti-angiogenic imbalance during pregnancy is associated with decreased myogenic tone, vasogenic edema, and an increase in BBB permeability, but not anti-angiogenic imbalance in the brain.

## 1. Introduction

Hemolysis elevated liver enzymes and low platelets (HELLP) syndrome affects 1 to 2 out of 1000 pregnant women in the United States per year, and occurs in 10–20% of women with preeclampsia with severe features [1,2]. Women with HELLP syndrome may experience hypertension, proteinuria, edema, headaches, visual disturbances, nausea, emesis, right upper quadrant pain, or midepigastric pain. The mechanisms responsible for the pathogenesis and pathophysiology of HELLP syndrome remain unclear. The manifestations of HELLP syndrome depend on the affected organ systems involved, which leads to the varying signs and symptoms of this disorder. The organ systems involved in HELLP syndrome include the renal, gastrointestinal, cardiovascular, pulmonary, and cerebrovascular systems. Central nervous system (CNS) involvement in HELLP syndrome is a major predictor of maternal morbidity and mortality [3,4]. CNS complications may include headaches unrelieved with acetaminophen, visual disturbances, vision loss, cerebral hemorrhage, cerebral infarction, cerebral edema, hypertensive encephalopathy, cerebral spasm, somnolence, and cognitive impairments [2,5]. Neurological complications have been reported in 50–66% of women with HELLP syndrome, and may occur in the antepartum, intrapartum, or postpartum period [6,7]. 

There is paucity in the literature regarding the mechanisms responsible for the CNS changes that occur in HELLP syndrome. HELLP syndrome is associated with changes in systemic inflammation, as characterized by immune system dysfunction, endothelial dysfunction, and angiogenic imbalance [8]. The systemic inflammatory changes that occur in HELLP syndrome may also play a role in the CNS abnormalities seen in women with HELLP syndrome, and may possibly contribute to the long-term vascular damage that has been reported in women with HELLP syndrome and severe preeclampsia [2,9,10,11]. We have previously reported that plasma from women with HELLP syndrome leads to impairment in the blood brain barrier (BBB), suggesting that yet undefined variables in the circulation system are contributing to the damage. 

To study these mechanisms, we infused anti-angiogenic factors soluble fms like tyrosine kinase 1 (sFlt-1) and soluble endoglin (sEng) into pregnant rats to create an animal model of HELLP syndrome [10,12]. Similar to what is reported in women with HELLP syndrome [13,14], animals with HELLP have evidence of increased inflammatory cytokines tumor necrosis factor-alpha (TNF-α), interleukin-17 (IL-17), and cytokine releasing CD4^+^ and CD8^+^ T cells [15]. These animals have increased Evans blue extravasation in the posterior cortex and brainstem/cerebellum region, as well as increased neuroinflammation and an increase in T cells [12]. However, when we pharmacologically blocked T cell activation and systemic inflammation in this model of HELLP syndrome, there was an improvement in BBB integrity which suggests peripheral inflammation directly contributes to BBB permeability [12]. However, the BBB is also affected by other factors, such as vascular tone, edema, and tight junction expression and function. All of these factors are potentially influenced by the inflammatory environment. Therefore, in the current study we tested the hypothesis animals with HELLP syndrome, which has a known systemic inflammatory state, have impairments in vascular tone, alterations in tight junction protein expression, and cerebral edema.

## 2. Methods

All studies were performed in 230–250 g timed-pregnant Sprague-Dawley rats (Envigo, Indianapolis, IN, USA). Animals were housed in a temperature controlled room with a 12:12 light/dark cycle. All experimental procedures used in this study were in accordance with the National Institutes of Health guidelines for use and care of animals, and were approved by the Institutional Animal Care and Use Committee at the University of Mississippi Medical Center. 

### 2.1. HELLP Rat Model

On day twelve of gestation (GD12), 4.7 µg/kg/day of recombinant mouse sFlt-1 Fc Chimera (soluble fms-like tyrosine kinase-1) and 7 µg/kg/day of recombinant rat sEng Fc Chimera (soluble endoglin; R&D systems, Minneapolis, MN, USA) were infused into the abdominal cavity of normal pregnant rats via mini-osmotic pumps (Model 2002; Alzet Scientific, Cupertino, CA, USA) to create HELLP syndrome for a period of 8 days [12,15,16]. Rats not infused with either sFlt-1 or sEng served as normal pregnant (NP) controls.

### 2.2. Measurement of Mean Arterial Pressure Conscious Rats and Determination of Hemolysis, Liver Enzymes, and Platelet Counts 

To determine mean arterial pressure (MAP), on GD18, rats underwent carotid artery catheter insertion under isoflurane anesthesia (Piramal Enterprises, Telangana, India) for blood pressure monitoring on GD19. The following day, pregnant rats were placed in individual restraining cages and allowed to acclimate to the restrainers for 30 min prior to arterial pressure recording. The pressure was recorded continuously for 30 min with a pressure transducer (Cobe III Transducer CDX Sema; Birmingham, AL, USA) and IWORX software (version 2.242000) connected to the indwelling carotid artery catheter, as previously described [15,17,18,19,20]. Immediately following MAP measurement, whole blood was collected in EDTA treated tubes to allow processing of lactate dehydrogenase (LDH), aspartate aminotransferase (AST), and platelets, and for sFlt-1 and sEng measurement [15]. Rats were euthanized while under isoflurane anesthesia and rat pups were collected and weighed. Brains were either used for myogenic response studies, cerebral edema studies, or isolated into frontal and posterior cortex, brainstem, and cerebellum, and frozen at −80 °C until further studies. 

### 2.3. Myogenic Response of Isolated Middle Cerebral Arteries (MCA)

On GD19, brains were removed and placed in ice-cold physiological saline solution (PSS; in mM—145 NaCl, 4 KCl, 1 MgCl_2_, 10 HEPES, 0.05 CaCl_2_, 10 glucose; pH 7.4). The MCAs were isolated from the brains and mounted on glass microcannulas and pressurized to 40 mmHg at 37 °C in an oxygenated (95% O_2_, 5% CO_2_) PSS bath and allowed to equilibrate for an hour. The inflow pipette was connected to a reservoir to allow control of intraluminal pressure that was monitored with a transducer (Cobe III Transducer CDX Sema; Birmingham, AL, USA), and the cannulated MCAs were visualized under a videomicroscopy system (model DRC; Zeiss, Oberkochen, Germany). The inner diameter of the vessels was measured using a videomicrometer (VIA-100; Boeckeler Instruments, Tuscon, AZ, USA). After preconditioning (2 sessions of intraluminal pressure increased from 40 mmHg to 140 mmHg), the inner diameter of the vessels was measured at intraluminal pressures from 40 to 140 mmHg in steps of 20 mmHg. After the pressure–diameter relationships was determined, the bath solution was replaced with Ca^2+^-free PSS and the passive pressure–diameter relationship was determined. The percent myogenic tone was calculated from the difference in diameter measured in Ca^2+^-free PSS and PSS divided by the diameter measured in Ca^2+^-free ×100.

### 2.4. Blood-Brain Barrier Permeability Studies

A separate group of rats (*n* = 5–6/group) was used to determine the extent of BBB permeability on GD19. First, 500 µL of 0.5 mg/mL of Texas Red Dextran (70,000 MW; Life Technologies, Carlsbad, CA, USA) reconstituted in Lactated Ringers solution (Life Technologies, Carlsbad, CA, USA) was infused via a carotid catheter into the rats and allowed to circulate for 10 min. Next, 500 µL of phenylephrine was infused step-wise every minute (5–500 µg) to induce acute hypertension. Animals were then perfused with Lactated Ringers solution to flush the dye from the cerebral circulation. Animals were then decapitated, and their brains removed and imaged using an In Vivo Imaging System (IVIS) (Perkin Elmer, Boston, MA, USA) with 595 nm excitation and 616 nm emission. 

Standards were prepared from the Texas Red solution and plated in duplicate at 100 µL/well in a 96-well plate. The regions of interest (ROI) were selected and applied to the selected brain regions (frontal cortex, posterior cortex, cerebellum, and brainstem), and the fluorescent radiant efficiency was determined for each brain region and fit to the standard curve. With the exception of the brainstem, the ROIs were uniform boxes in fixed positions utilized for all rats. If the fixed ROI brainstem box was not completely filled with a brainstem sample, the sample was not used for analysis. This was the case with one of the NP brainstem samples. The mean standard fluorescent intensity was calculated per region per rat, and for all rats in each group. Both the brains and the plate of standards were imaged using Living Image 4.3 software (Perkin Elmer, Boston, MA, USA). 

### 2.5. Evaluation of Occludin Protein Expression via Western Blot

To determine the quantity of occludin expression in the posterior cortex and brainstem of HELLP and NP rats, western blots were performed on these brain sections (*n* = 3/group). Samples were mechanically homogenized in homogenization buffer (10 mM Tris base (Fisher-Scientific) with 37.22 mg Ethylenediaminetetraacetic acid (EDTA; Sigma–Aldrich, St. Louis, MO, USA), diluted in 100 mL distilled water, pH 7) containing protease inhibitor cocktail III, Mammalian (Research Products International, Mount Prospect, IL, USA) on ice. The homogenate was centrifuged for 10 min at 1600 RPM and 4 °C, and the supernatant collected. A bicinchoninic acid protein assay (BCA; ThermoFisher, Rockford, IL, USA) was performed using standard protocol to obtain the protein concentration. 

The samples were mixed with sample buffer (125 mM Tris base, 20% glycerol (Sigma–Aldrich,), 4% sodium dodecyl sulfate solution (SDS, Bio-Rad, Hercules, CA, USA), 10% mercaptoethanol (Sigma–Aldrich, St. Louis, MO, USA), 0.05% bromophenol blue (Sigma–Aldrich), pH 6.8) and heated at 95 °C for 10 min. Next, 50 µg of the proteins was electrophoresed (BioRad, Hercules, CA, USA) and transferred onto nitrocellulose membranes followed by immersion in Ponceau-S stain (Sigma–Aldrich, St. Louis, MO, USA)) to ensure protein transfer. After the membranes were destained, they were blocked with 5% milk buffer, and washed and incubated overnight at 4 °C with 1:750 mouse anti-occludin monoclonal antibody (BD Transduction Laboratories, Franklin Lakes, NJ, USA) in 5% milk buffer with Tween 20. The following day, after washing, membranes were incubated with 1:2500 goat anti-mouse IgG horseradish peroxidase (HRP, Santa Cruz Biotechnology, Dallas, TX, USA) and 1:5000 Strep Tactin HRP conjugate (BioRad, Hercules, CA, USA) for one hour on a shaker at room temperature before being developed with Clarity Western enhanced chemiluminescence (ECL) substrate (BioRad, Hercules, CA, USA). The membrane was scanned using the Chemidoc^™^ XRS+ system (BioRad, Hercules, CA, USA). The bands were analyzed using Image J software, which is a java-based image analysis program developed at the U.S. National Institutes of Health. Image J converts digital images to 8-, 16-, and 32-bit pictures, and calculates the area and pixel value based on user-defined threshold intensity [21]. 

### 2.6. Determination of Brain Water Content

On GD19, brains (*n* = 4/group) were removed, dissected into frontal and posterior cortex, brainstem, and cerebellum, and the wet weight recorded. Brain sections were dried in an oven at 95 °C for 24 h, and reweighed to obtain the dry weight. Brain water content was calculated as ((wet weight−dry weight)/wet weight) × 100.

Brains were collected from a separate group of rats (*n* = 3/group) on GD19 and fixed in 10% buffered formalin, embedded in paraffin, and cut into 4 µM sections. Two sections per animal were stained by hematoxylin and eosin (H&E), and two areas of the posterior cortex per section were examined. 

### 2.7. Circulating and Brain Levels of sFlt-1 and sEng

Plasma and aliquots of the brains homogenized above were used to measure levels of sFlt-1 and sEng via commercially available enzyme linked immunosorbent assays, according to the manufacturer’s protocol (R&D Systems, Minneapolis, MN, USA). The mouse sFlt-1 ELISA displayed a sensitivity of 3.5 pg/mL, interassay variability of 7.3%, and intra-assay variability of 3.6%. The mouse sEng ELISA displayed a sensitivity of 13.6 pg/mL, interassay variability of 6.7%, and intra-assay variability of 4.2%. All samples were assayed in duplicate (2 wells per sample).

### 2.8. Statistical Analysis

Data are expressed as mean ± standard error mean and were analyzed via Student’s *T* test using GraphPad Prism 7.02. Repeated measures two-way analysis of variance was used to determine if there was a statistically significant interaction between groups in lumen diameters in the myogenic response experiments. Data were considered statistically significant if *p* < 0.05.

## 3. Results

### 3.1. Mean Arterial Pressure and Symptomology of HELLP Syndrome Occur in Response to Infusion of sFlt-1 and sEng during Pregnancy

**Figure 1:**
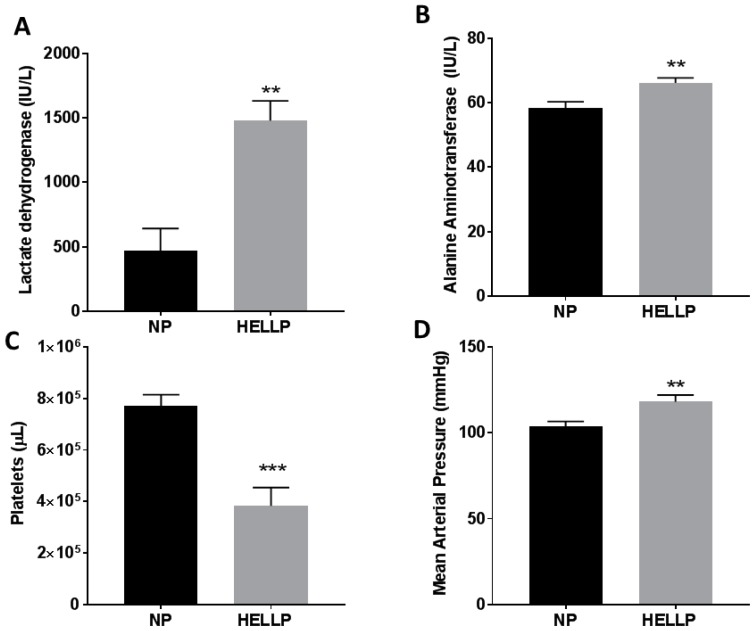
The biochemical factors associated with hemolysis elevated liver enzymes and low platelets (HELLP) syndrome were significantly increased and decreased, respectively, when sFlt-1 and sEndoglin were infused into normal pregnant (NP) rats. Hemolysis (measured by lactated dehydrogenase, (**A**)) and liver enzymes (alanine aminotransferase, (**B**)) were significantly increased compared to NP rats, and platelets were significantly decreased (**C**). Mean arterial pressure was also significantly increased in HELLP rats when measured via carotid catheter at gestational day 19 (**D**). **, *** denotes *p* < 0.05, 0.001 compared to NP. *n* = 16/group.

As we have previously reported, infusion of sFlt-1 and sEng during pregnancy significantly increases hemolysis (*p* = 0.003), liver enzymes (*p* = 0.006), and decreases platelets (*p* < 0.0001) compared to NP rats (Figure 1A–C). These changes also occur in conjunction with a significant increase in blood pressure when MAP is measured on GD19 (*p* = 0.007, Figure 1D). There were no statistically significant differences in pup weight between the NP and HELLP rats (2.48 ± 0.03 g vs. 2.44 ± 0.05 g, *p* = 0.54). 

### 3.2. Myogenic Tone and BBB Are Impaired in Response to HELLP Syndrome

**Figure 2:**
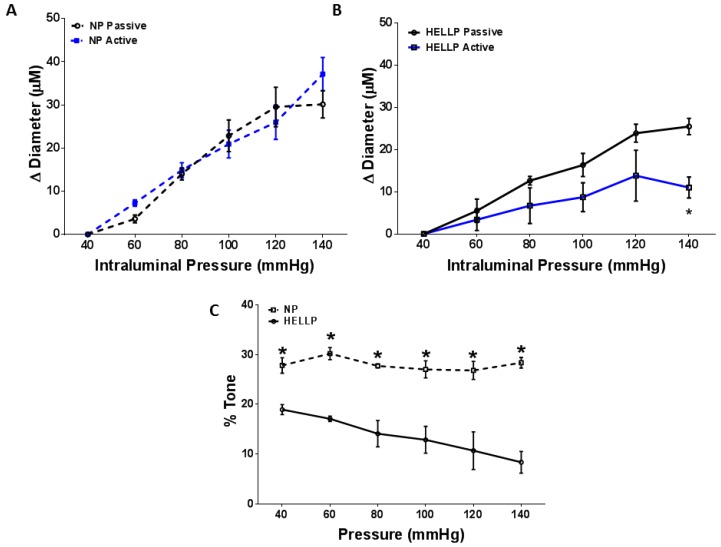
The middle cerebral artery (MCA) was isolated from normal pregnant and HELLP rats, and the relationship in changes to the lumen diameter between the active (with Calcium) and passive (without Calcium) responses was analyzed for NP (**A**) and HELLP (**B**) rats. The myogenic tone of the vessels was calculated and found to be significantly different at all pressures between NP and HELLP rats (**C**). * denotes *p* < 0.05 compared to the opposite data point at the same pressure. NP, *n* = 4; HELLP, *n* = 3.

Experiments were performed to determine if the myogenic response was impaired in rats with HELLP syndrome. There was not a significant difference in MCA lumen diameter in NP rats between active and passive responses (*p* = 0.41, Figure 2A). However, when we compared the active and passive responses of HELLP rats, there was a statistically significant difference between the responses (*p* = 0.02, Figure 2B). The percent tone vs. pressure for MCAs from NP and HELLP rats is shown in Figure 2C. There was a statistically significant relationship (*p* = 0.02) between the groups at each pressure point, suggesting that rats with HELLP syndrome had a decrease in myogenic tone compared to NP rats.

**Figure 3:**
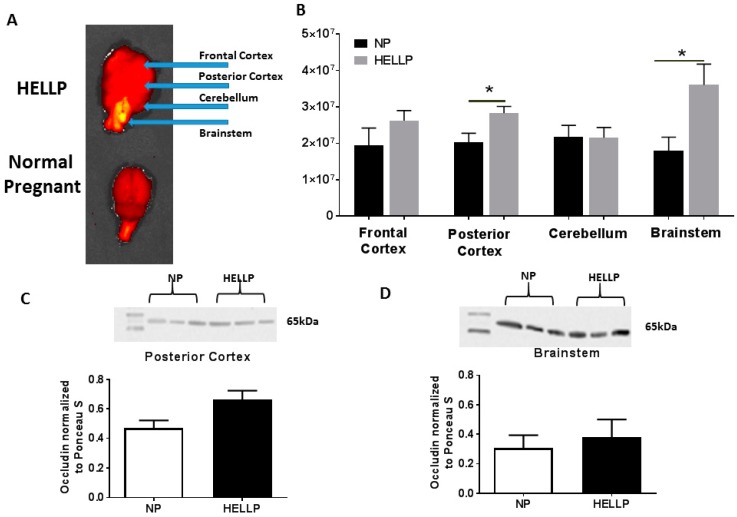
A representative NP and HELLP brain from rats infused with 0.5 mg/mL of Texas Red and phenylephrine to detect blood brain barrier (BBB) structural integrity (**A**). The blue arrows indicate the regions of interest that were analyzed utilizing In Vivo Imaging System (IVIS). HELLP rats had significantly more Texas Red in the posterior cortex and in the brainstem compared to NP rats (**B**). Representative western blot of homogenates from the posterior cortex (**C**) and brainstem (**D**) depicting the characteristic band at 65 kDa for occludin, and bar graphs showing the relationship between NP and HELLP rats within each respective brain region. Data are represented as mean ± SEM; * denotes *p* < 0.05; NP, *n* = 3–5; HELLP, *n* = 3–6.

When we evaluated BBB in the frontal cortex, there was not a statistically significant difference between NP and HELLP rats (*p* = 0.25); however, Texas Red fluorescence was significantly increased in the posterior cortex (*p* = 0.03, Figure 3B). In the cerebellum there was not a statistically significant difference between the NP and HELLP rats (*p* = 0.97). There was significantly more Texas Red in the brainstems of HELLP rats compared to those of NP rats (*p* = 0.05, Figure 3B). 

### 3.3. HELLP Syndrome Increases Brain Water Content in the Posterior Cortex

As IVIS imaging revealed that BBB permeability was significantly increased in the posterior cortex and the brainstem of HELLP rats, we performed a western blot for the tight junction protein occludin in these two regions of the brain. There was not a statistically significant difference in occludin expression in the posterior cortex (0.47 ± 0.05 vs. 0.66 ± 0.06 arbitrary units, *p* = 0.08, Figure 3C) or in the brainstem (0.38 ± 0.12 vs. 0.30 ± 0.09 arbitrary units, *p* = 0.65, Figure 3D) between NP and HELLP rats. 

**Figure 4:**
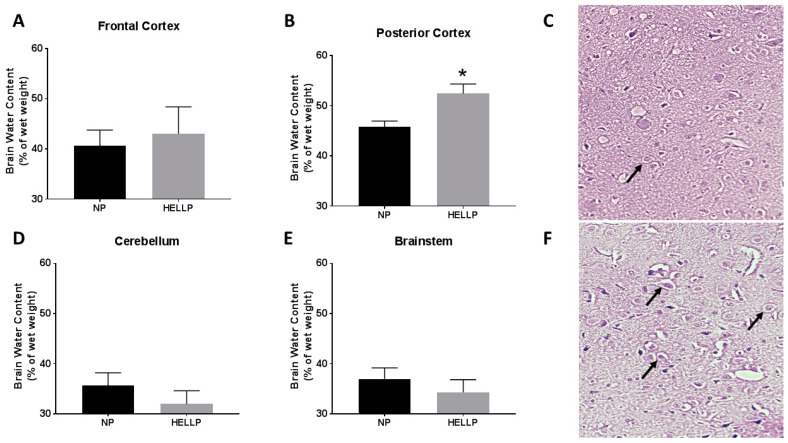
Brains were sectioned into frontal cortex, posterior cortex, cerebellum, and brainstem, and brain water content was measured. There was not a statistically significant difference in water content in the frontal cortex (**A**), however rats with HELLP syndrome had significantly more water content in the posterior cortex (**B**). There were no statistically significant changes in the cerebellum (**D**) and the brainstem (**E**). Light microscopy of the posterior cortex from NP control rats (**C**), and HELLP (**F**) rats with evidence of mild cerebral edema, leukocyte infiltration, and perivascular edema, as indicated by the black arrows—20× Magnification. Data are represented as mean ± SEM; * denotes *p* < 0.05; *n* = 3–4/group.

Brain water content was measured in the frontal and posterior cortex, as well as in the cerebellum and brainstem. There was not a statistically significant difference in water content in the frontal cortex (*p* = 0.72, Figure 4A), cerebellum (*p* = 0.36, Figure 4D), or brainstem (*p* = 0.46, Figure 4E) between NP and HELLP rats. However, brain water content in the posterior cortex was significantly increased in HELLP rats compared to NP rats (*p* = 0.02, Figure 4B). We next examined if there was a correlation between MAP and brain water content in the posterior cortex to determine if these changes could be associated with hypertension, and we did not find a significant association between the two (*r* = 0.20, 95% CI: 0.59–0.79, *p* = 0.63).

The pathology of the NP brains showed normal cytoarchitecture and perivascular swelling (Figure 4C). Sections from HELLP rats had evidence of leukocyte infiltration and mild cerebral edema (Figure 4F).

Circulating levels of sFlt-1 were significantly increased in HELLP (4480 ± 320.2 pg/mL) compared to NP rats (3071 ± 364.2 pg/mL, *p* = 0.01). Similar results were seen in circulating levels of sEng in which HELLP rats (72.64 ± 6.5 pg/mL) had significantly higher levels of sEng compared to NP rats (41.35 ± 5.5 ng/mL, *p* = 0.003). Finally, we measured sFlt-1 and sEng protein expression in the posterior cortex and the brainstem of NP and HELLP rats. Neither sFlt-1 (1177 ± 124.7 vs. 1124 ± 107 pg/mg/mL, *p* = 0.76) nor sEng (2.51 ± 0.51 vs. 1.95 ± 0.44 ng/mg/mL, *p* = 0.43) were significantly increased in the posterior cortex between the groups. In the brainstem, sFlt-1 (757.1 ± 98.76 vs. 714.6 ± 44.9 pg/mg/mL, *p* = 0.71) was not significantly different between NP and HELLP rats, however sEng was significantly decreased in rats with HELLP (2.2 ± 1.3 pg/mg/mL) compared to NP rats (8.51 ± 1.3 pg/mg/mL, *p* = 0.01).

## 4. Conclusions

HELLP syndrome is a serious obstetric disorder with associated maternal and fetal morbidity and mortality, including cerebral complications [11]. Cerebrovascular involvement seen in preeclampsia and eclampsia is a leading cause of maternal mortality [22]. There is paucity in the literature regarding the mechanisms responsible for the cerebral complications that occur in HELLP syndrome. While we have previously reported that infusion of sFlt-1 and sEng into normal pregnant rats induces HELLP syndrome and increases systemic inflammation, endothelial activation, oxidative stress, BBB permeability, and neuroinflammation, we did not investigate the possible mechanisms of these neural changes [15,16,23]. In the current study, we have new data indicating that (1) myogenic tone is decreased in the middle cerebral arteries of HELLP rats, and (2) HELLP rats have cerebral edema. Additionally, we were able to repeat our previous studies which have shown that rats with HELLP syndrome have a regional selectivity for BBB disruption in the posterior cortex and brainstem.

Impairments in the myogenic response can lead to changes in the autoregulation of cerebral blood flow, and ultimately contribute to cerebrovascular disturbances such as BBB disruption and cerebral edema [24]. In the current study we report that myogenic tone is impaired in MCAs isolated from HELLP rats compared to NP rats. These results are in agreement with previous results by Ryan et al., who reported in the reduced uterine perfusion pressure (RUPP) model of placental ischemia (preeclampsia animal model) that myogenic tone is impaired in the MCA [25]. As we have previously reported no change in myogenic reactivity between women with a normal pregnancy and those with HELLP syndrome when their plasma was infused into posterior cerebral arteries (PCA) and myogenic reactivity measured [10], we examined the MCA in the current study. Interestingly, a study by Amburgey et al. reported similar results when plasma from women with preeclampsia was used in the PCA [26]. It could perhaps be that the PCA is not an adequate model to examine ex vivo myogenic reactivity, or that circulating factors in women with HELLP syndrome and preeclampsia do not induce myogenic reactivity in the PCA. This is supported by the fact that myogenic studies during pregnancy using the cerebral vein of Galen or parenchymal arterioles both report a decrease in myogenic tone in response to pregnancy [27,28]. 

Consequently, the RUPP animal model, which has an impaired myogenic response, also has been reported to have evidence of impairments in cerebral autoregulation and BBB permeability [29,30]. Cerebral autoregulation in the current model of HELLP syndrome has not been measured, but other animal models of hypertension during pregnancy [31,32] have reported alterations in cerebral autoregulation, as well as studies examining patients with preeclampsia and HELLP syndrome [33,34]. A study by Zunker et al. measured blood flow in women with pre-eclampsia and/or eclampsia and reported increased blood flow in both the MCA and PCA, whereas of the women with HELLP syndrome who had an increase in blood flow (5/11), there was only an increase in the MCA [35]. As the MCA supplies blood primarily to the frontal, temporal, and parietal lobes (the PCA primarily serves the hippocampus, occipital lobes, and the lower areas of the temporal lobe), damage or edema to the frontal cortex may have been expected instead of the increased edema and BBB permeability in the posterior cortex that we report here. We would need to measure cerebral blood flow to determine if a loss of myogenic tone in the frontal cortex increases cerebral blood flow, which could potentially account for the edema and increased BBB disruption seen in the latter part of the brain [36].

In the current study, we were unable to demonstrate an increase in BBB permeability in the frontal cortex of HELLP rats relative to NP rats after phenylephrine administration. However, in agreement with previous findings, we did see a significant increase in BBB permeability in the posterior cortex and brainstem of HELLP rats [12]. Both the posterior cortex and the brainstem are targeted in women with HELLP syndrome and preeclampsia, as well as in the RUPP animal model of placental ischemia [7,37,38]. Furthermore, we have recently reported that more than six weeks after pregnancy, rats infused with sFlt-1 and sEng during pregnancy remained hypertensive and still had significant BBB damage in the posterior cortex and brainstem compared to post-partum NP rats, suggesting that not only are these regions of the brain susceptible to damage during pregnancy, but that the effects are long-lasting [39]. 

We also evaluated the tight junction protein occludin in the posterior cortex and brainstem, as these two regions of the brain had evidence of BBB compromise both in the current study and in our previous study [12]. While a reduction in occludin expression could have served as a possible mechanism or contributor to the increase in BBB permeability, it was not altered in response to anti-angiogenic imbalance during pregnancy in either the posterior cortex or the brainstem. Similar results have been reported in both animal models of hypertension and in the RUPP animal model [29,40]. In these studies, there were not any differences in occludin, claudin, and zona-occludin expression between control and experimental models, suggesting that perhaps an alteration of tight junction proteins may not be related to BBB impairment during pregnancy. Additionally, studies examining the murine blood-retinal-barrier found that overexpression of sEng results in an increase in the Evans blue extravasation and a decrease in occludin protein expression, pointing to a negative association between barrier function maintenance and sEng [41]. 

Localized cerebral edema was present in the posterior cortex of rats with HELLP syndrome. Evidence of vasogenic edema (edema associated with BBB disruption resulting in an increase in extracellular space) was further confirmed by histopathology in the posterior cortex of HELLP rats [42]. Studies in women with preeclampsia, HELLP syndrome, or eclampsia have found that the posterior regions of the brain are more susceptible to edema formation compared to other regions [7,43]. While there was not a significant association between MAP and cerebral edema in the current study, other animal models of acute hypertension during pregnancy have reported cerebral edema and BBB disruption in both the anterior and posterior regions of the brain compared to non-pregnant rats [32,40]. Important to our studies and those of others, is the fact that similar to what is seen in women with different diagnoses of hypertensive disorders (preeclampsia vs. eclampsia vs. HELLP syndrome), each animal disease or disorder animal model has different neurological outcomes.

Though we did not measure peripheral levels of inflammatory markers in the current study, we have previously reported that chronic infusion of sFlt-1 and sEng increases circulating levels of IL-17, TNF, and IL-6, as well as contributes to neuroinflammation [12]. As expected and previously published, we did find that circulating levels of both sFlt-1 and sEng were significantly increased in HELLP rats compared to NP rats [12,15,23]. At this point we have new data suggesting there is not an anti-angiogenic imbalance (sFlt-1 and sEng) in the maternal brain in HELLP rats, suggesting the neuroinflammation present in HELLP rats may be due to downstream effects of the peripheral angiogenic imbalance.

Over the past few years, there have been a number of studies reporting that not only is the pregnant brain different, but that hypertension and placental ischemia significantly impact neuroinflammation, cerebral edema, cerebral autoregulation, and BBB permeability [15,38,44,45]. Recently, we have used an anti-angiogenic model of HELLP syndrome to demonstrate that in the absence of severe hypertension, rats with HELLP syndrome have increased BBB permeability and neuroinflammation [12]. Now, in the current study we are reporting that these rats also have cerebral edema and impairment in myogenic tone. Though the present studies are focused on the effect of angiogenic imbalance on the maternal brain during pregnancy, similar results have been reported with sFlt-1 and sEng infusion in non-pregnant animals. When sFlt-1 and sEng were overexpressed in mice, BBB permeability was increased and periventricular edema developed [46]. Importantly, these changes did not occur in animals that received only sFlt-1 or sEng, but rather the combination of both. 

One major limitation in the current study is the lack of a mini-osmotic pump SHAM group to determine the effect of inflammation associated with surgery performed on GD12 and the effect of the extra volume (the mini-osmotic pump releases approximately 0.5 ± 0.1 µL/h) on the assays performed on GD19. However, as previous studies have reported that pregnant SHAM rats undergoing a midline abdominal incision on GD14 had no evidence of neuroinflammation on GD20 [38], it is likely that any inflammation associated with the abdominal surgery conducted early in pregnancy does not have an adverse effect on the brain physiology. However, as that study did not utilize mini-osmotic pumps, future studies will need to include a SHAM mini-osmotic pump group to ensure the extra fluid volume that is being infused from the mini-osmotic pump is not contributing to cerebral edema, inflammation, or any of the other variables within our model.

In summary, when sFlt-1 and sEng are increased to supra-physiologic levels, such as that seen in women with severe preeclampsia and HELLP syndrome [8], there is selective targeting of the posterior cortex and the brainstem. The combination of these studies begins to paint a pathophysiologic picture supporting the hypothesis that hypertension and systemic inflammation during pregnancy contributes to neurological complications, which moving forward will allow increased therapeutic and mechanistic studies to be developed.
